# Gender-Disaggregated Consumer Testing and Descriptive Sensory Analysis of Local and New Yam Varieties

**DOI:** 10.3390/foods12030537

**Published:** 2023-01-25

**Authors:** Liticia Effah-Manu, Faustina D. Wireko-Manu, Jacob K. Agbenorhevi, Busie Maziya-Dixon, Ibok N. Oduro

**Affiliations:** 1 Department of Food Science and Technology, Ho Technical University, Ho P.O. Box HP 217, Ghana; 2 Department of Food Science and Technology, Kwame Nkrumah University of Science and Technology, PMB KNUST, Kumasi AK-039-5028, Ghana; 3 Postharvest and Nutrition Laboratory, International Institute of Tropical Agriculture (IITA), PMB 5320, Ibadan 200001, Nigeria

**Keywords:** gender-disaggregated, descriptors, quality, yam, descriptive testing, consumer testing

## Abstract

Gender-disaggregated sensory evaluation has become an essential element that could enhance breeding activities by increasing the adoption of new varieties. The effect of age, sex and geographical location on descriptor preferences for boiled and pounded yam were studied using descriptive and consumer testing. Attributes with definitions and measurement scales were used to generate lexicons for boiled and pounded yam. Analytical tools employed for the inferential statistics were the independent *t*-test, analysis of variance, Kruskal–Wallis test, Mann–Whitney test and relative importance index (RII). Descriptive testing showed that all the *D. rotundata* varieties were good for boiling and pounding. The *D. alata* varieties *afase soanyinto* and *afase biri* were most liked, while *afase pa* and *ahodenfo* were disliked. Age had no significant effect on descriptor preferences. Being a female or male, however, influenced preferences for pounded yam descriptors such as mouldability, lumpiness and colour. The RII for the *D. rotundata* varieties (0.22–0.28) showed that they are all good varieties for boiling and pounding. The local *D. alata* varieties were still highly acceptable compared the new CRI varieties due to the aroma. Rural consumers preferred all the descriptors of boiled *D. rotundata* than urban consumers, whereas urban consumers liked the pounded yam varieties better than the rural consumers. Availability of the new yam varieties on local markets could therefore increase consumption and improve adoption.

## 1. Introduction 

Yam is an important staple in the humid and semihumid tropical Asia and Americas. According to Maroya et al. [1], yam is the second most important root/tuber crop after cassava in terms of providing food security in Africa. In yam-consuming countries, it serves as a good source of carbohydrates, and adds more than 200 dietary calories per capita daily, according to Thompson and Onyenweaku [2].

Environmental changes that bring about stressful conditions in yam production make it more important to breed new varieties that could withstand them. When new varieties are released, sensory evaluation is the main assessment that needs to be carried out on them. Sensory evaluation is defined by Stone et al. [3] as a scientific method that evokes, measures, analyses and interprets responses to products, as perceived through the senses of sight, aroma, touch, taste and sound. Stone and Sidel [4] grouped sensory evaluation into affective and analytical tests. In affective testing, a large number of untrained panellists are required to represent the target population. Analytical tests, on the other hand, are used for the laboratory evaluation of products and for the identification and quantification of sensory characteristics. The major examples of this test are discrimination and descriptive. 

According to Stone and Sidel [4], descriptive tests require a trained panel, but the participants in a discrimination test need to be partly trained or sometimes untrained. Work conducted by Hampson et al. [5] shows that descriptive sensory evaluation is the most informative and comprehensive as it gives detailed information, and is the standard for sensory characterization. According to the authors, more reliable data could be generated by breeders when formal sensory evaluation is applied. In acceptance testing, the degree of liking is rated, while the preference identifies the product that is liked more usually using a category scale (e.g., hedonic scale). Consumer acceptability tests, however, use a scaling method to measure the degree of liking or disliking of a product. This test is the most important, and must be conducted when new varieties are released. Hampson et al. [5] further reiterates that food preference is influenced by sensory perception, which affects acceptance of food commodities.

Accepting a particular food product or new food crop may be affected by a number of factors, including culture, attitudes and beliefs, as indicated by Köster [6]. Rappoport et al. [7] also found that age and gender influence food preferences. However, it has been indicated by Kittler et al. [8] that food selection is primarily stimulated by personal taste (colour, aroma and texture). Studies related to sensory descriptive analysis and consumer tests have been extensively carried out by Lestringant et al. [9]. Nonetheless, there is limited literature concerning gender-disaggregated data of preferences for quality descriptors of yam. According to Mudege et al. [10], this will enhance gender-responsive breeding, which means “providing farmers with a basket of choices so that traits that men and women value are included during product profile development”. 

Therefore, the objective of the present study was to use descriptive testing to ascertain the descriptors of quality of boiled and pounded yam from both new and local varieties and to determine the consumer preferences for the descriptors of quality as influenced by sex, age and community.

## 2. Materials and Method

### 2.1. Population Characteristics and Sample for Consumer Testing

The Brong Ahafo and the Northern Regions of Ghana are noted for high yam production. For the purposes of this work, the Brong Ahafo Region (Now Bono East) was selected for the study based on the high production of (39%) compared to 25% for the Northern Region, as stated by SRID [11]. The region lies between longitude 00° 15” E-1° 30” W 00 15′ E-30 W and latitude 8° 45” N-7 30” N 80 45′ N-70 30′ with an estimated population of 2,282,128. Data from the Ghana Statistical Service [12] give the population of Sene West District, Kintampo Mmunicipality and Techiman East District to be 57,734, 95,480 and 59,068, respectively.

The same communities used for the survey aspect of the work by Effah-Manu et al. [13] were used for the consumer testing. A total of twelve (12) communities made up of three district capitals from each selected district and nine other communities (three from each district) were used. Sixty (60) respondents in each of the district capitals and twenty (20) from each of the other communities were used. In total, 360 respondents (120 from each of the district) participated in the consumer testing. The ratio of female to male was 1:1 for all the communities. 

### 2.2. Acquisition of Yam

New yam varieties were obtained from the Crops Research Institute (CRI) in Fumesua, Kumasi-Ghana. Local varieties were obtained from a known farmer at Mampong in the Ashanti Region of Ghana. All yam samples used in the study were stored for 3 months after harvest, but COVID-19 restrictions lengthened the storage of *D. rotundata* varieties. The yams were stored in yam barns at the CRI-Fumesua until they were ready for use.

### 2.3. Preparation of Boiled and Pounded Yam

Yams were peeled and cut manually into 2 × 2 cm cubes and cooked in boiling water in a saucepan with 180 mL of water under high-heat intensity gas oven (T/SZCX 002-2018, China) for approximately 15 min and 20 min, respectively, for *D. alata* and *D. rotundatata* varieties based on preliminary work. Boiled yam was kept in plastic bowls with cover and placed in a food warmer at approximately 70 °C for sensory evaluation. Pounded yam was prepared from the boiled yam using a commercial *fufu* pounding machine (GH X21, Enebel Fabricators, Ghana 2015). For all the varieties, samples were extruded three times to obtain a consistent product for analysis without adding water.

### 2.4. Panel Training and Descriptive Testing

An initial panel of 20 was recruited from the Kwame Nkrumah University of Science and Technology (KNUST), Kumasi and trained for three weeks. Panel training was conducted by the researcher, who had received sensory panel training by RTB Foods and CIRAD at the Nairobi Agricultural Research Laboratories, Kawanda in 2018. Training included basic acuity tests, i.e., identification of sweet, sour and bitter using sugar, lime and quinine. Threshold studies and identification of aromas were also conducted. Panellists were further trained on triangle and difference tests. During this period, panel members whose scores were extremely below or above the panel average were eliminated. Eventually, a 12-member panel was constituted and trained in identification of descriptors of quality of boiled and pounded yam. After ensuring that panellists’ scores for the various attributes were good using XLSTAT 2019.4, they were oriented on how to generate attributes for the samples. The panel generated attributes with definitions and how to measure and scale for measurement, and later used the lexicon generated as a guide to analyse boiled and pounded yam samples. Eleven yam varieties of *D. alata* and *D. rotundata* made up of four local and seven unspecified CRI (new) varieties with varying sensory characteristics were prepared and served to the panel.

The descriptive testing was conducted in the Sensory Laboratory of the Department of Food Science and Technology, KNUST-Kumasi. The panel was made up of five males and seven females. Prepared samples were served in transparent plastic containers with cover lids. Pounded yam was served at room temperature while boiled yam was served hot. Samples were blind-labelled with random three-digit codes in a randomized order using XLSTAT 2019.4 for each serving. The room was well-lit and each panellist was provided with a booth, the sample and water for palate cleansing. To avoid the effect of sample size and tuber part variations on textural attributes, panellists were served with approximately the same sample size and shape as well as the tail, middle and head part of yam.

### 2.5. Consumer Testing 

People who are regular consumers of boiled and pounded yam from the different yam species (*D. alata* and *D. rotundata*) were used for the consumer testing. Areas for consumer testing were carefully selected to ensure that physical and chemical factors did not interfere with the procedure. Hence, community centres, areas around market centres and church auditoriums were used for the test. The holding time, defined as the minimum and maximum time after preparation that a product can be used for sensory test, was considered. Therefore, testing was conducted within at most 1 h for each set of respondents. Samples were kept in a food warmer during the holding time to ensure consistency in test results. Samples were blind-labelled with random three-digit codes in a randomized order using (XLSTATS 2019.4) for each serving. Transparent containers with lids were used for the presentation. Water was used as palate cleanser to aid in the removal of residues from previous samples. 

### 2.6. Statistical Analysis 

Statistical package for social science (IBM SPSS Statistics for windows, Version 23.0. Armonk, NY:IBM Corp.) (SPSS, Version 23) was used for the analysis of both the descriptive and consumer testing. Average scores, as well as overall liking of test varieties in descriptive and consumer testing, were calculated using ANOVA. Principal component analysis (PCA) was used to relate/link the various descriptors to the varieties that best possessed them. Independent *t*-test was used to test the significance between two sample means. Kruskal–Wallis test was used to test and compare the effect of age group of participants on descriptor preferences. In ranking the varieties based on the preferences of the descriptors, the relative importance index (RII) was used, and Mann–Whitney U test was employed to establish the variations in descriptor preferences among males and females. Significance was determined at *p* < 0.5.

### 2.7. Ethical Considerations

Participation in this research was fully voluntary. The study ensured that key principles such as informed consent and anonymity outlined in the Humanities and Social Sciences Research (HuSSR) Policy of the Kwame Nkrumah University of Science and Technology [14] were followed. 

## 3. Results and Discussion 

### 3.1. Descriptors of Quality of Boiled and Pounded Yam as Identified by Descriptive Sensory Panel

The quality descriptors from the panel (Table 1a,b) are similar to those reported by Otegbayo et al. [15] and Akissoe et al. [16]. For pounded yam, Otegbayo et al. [15] reported fibrousness (i.e., presence of fibrous strands) as a quality descriptor. However, during the deliberations of the panel, it was noted that most commonly consumed yams in Ghana do not have this quality descriptor, so they agreed to exclude it. Similarly in the survey work by Effah-Manu et al. [13], heaviness for boiled yam and shelf life for pounded yam, which are not directly/physically seen on the food product, were included as descriptors. Further, during the preliminary test of the consumer testing questionnaire, respondents suggested the removal of the descriptors of fracturability and stickiness. They argued that it was difficult to understand fracturability as it is closely related to mouldability. For stickiness, they noted that it is not an important descriptor as at the point of eating, the presence of soup (usually liquid in Ghana) makes it difficult to consider this descriptor. These suggest that the descriptors of quality of boiled and pounded yam are country-specific. Although considered difficult to score, these two attributes were still kept within the lexicon.

The means of descriptors from the scoring are found in Table 2. For boiled *D. alata* varieties, local variety, *matches* had highest value for whiteness while *akaba* reported the highest aroma value. In terms of taste, only *ahodenfo* came close to the local varieties. However, mealiness values were high and compared well with local varieties. For *D. rotundata* boiled yam varieties, values for many descriptors are quite related, indicating that descriptors of quality are liked almost in the same manner as local varieties.

On the other hand, average values for colour, aroma, stretchability and lumpiness for all the CRI *D. alata* varieties were lower than the local varieties for pounded yam samples. Average scores for aroma and colour of the CRI *D. rotundata* yam varieties were approximately equal to the local varieties, indicating high preference for these descriptors. Generally, the CRI *D. rotundata* compared favourably with counterpart local varieties.

### 3.2. Principal Component Analysis (PCA) of Descriptors of Quality of Boiled and Pounded Yam Samples

**Figure 1:**
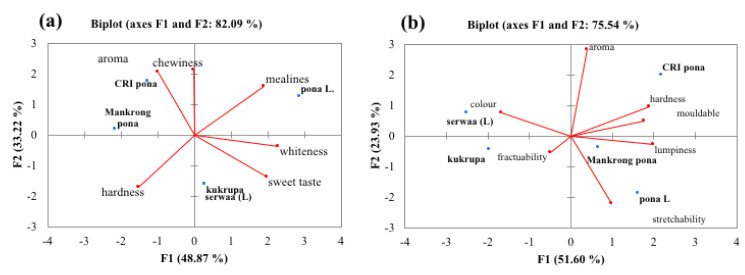
Principal component of descriptors of quality for (**a**) boiled and (**b**) pounded *D. rotundata* varieties. Yam varieties: *CRI pona, CRI kukrupa, CRI Mankrong pona, serwaa, local pona*; L = local variety.

The PCA was applied to data from descriptive analysis of boiled and pounded yam to find out how the different varieties associate with the various descriptors of quality. F1 and F2 together explain 82% of variance observed in the data for boiled yam (Figure 1a). In terms of mealiness, local *pona* was most mealy, while *CRI pona* had a good aroma and was chewy. *Mankrong pona* and *serwaa* were found in-between sweet taste and hardness. For pounded yam (Figure 1b), *kukrupa* was found to be most fracturable. Aroma, mouldability and preferred hardness were characteristics of CRI *pona*, whilst the local *pona* was most stretchable. F1 and F2 explain the 75.54% of variability in data.

For the boiled samples, *afase soanyinto* was hardest. In terms of the descriptors of sweet taste and aroma, all the varieties were found farther, except *afase biri* and *akaba*, which were close to having sweet taste. These same varieties were also seen as highly chewy. However, *afase soanyinto*, *akaba* and *afase biri* were slightly close to the descriptor of mealiness. *Afase ahodenfo* and *afase pa* were seen farther from all the descriptors of quality of boiled yam. This clearly indicates that these varieties are not suitable for boiled yam.

**Figure 2:**
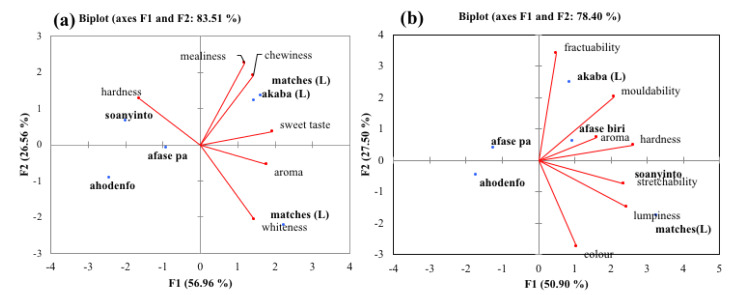
Principal component of descriptors of quality for (**a**) boiled and (**b**) pounded *D. alata* varieties. Yam varieties—*CRI afase pa, CRI afase biri, CRI afase pa, CRI ahodenfo, CRI soanyinto, akaba matches*. L = local variety.

Figure 2b indicates that *afase pa* and *afase ahodenfo* do not qualify as poundable varieties. This is because they are found opposite to the descriptors of quality for pounded yam. *Afase biri* had a high pounded yam aroma than the rest of the varieties. *Afase soanyinto* (CRI) and *matches* (local) were found to be stretchable and lumpy, respectively. *Akaba* was the variety that was very white compared to the others. 

The results from texture studies by Effah-manu et al. [17] indicated that the new *D. alata* varieties *afase soanyinto* and *afase biri* are good varieties for boiled and pounded yam as seen in the PCA for descriptive testing. According to Otegbayo et al. [18], dry matter content, starch and amylose rations may be responsible for good cooking quality of boiled yam. For *Afase soanyinto*, the starch content (17.0%), which is similar to that of the *D. rotundata* varieties (17–20.5%) as determined by Effah-Manu et al. [19], may have accounted for its superior characteristics. 

### 3.3. Effect of Sex of Respondents on Descriptor Preferences for Boiled D. alata and D. rotundata Yam Varieties

**Figure 3:**
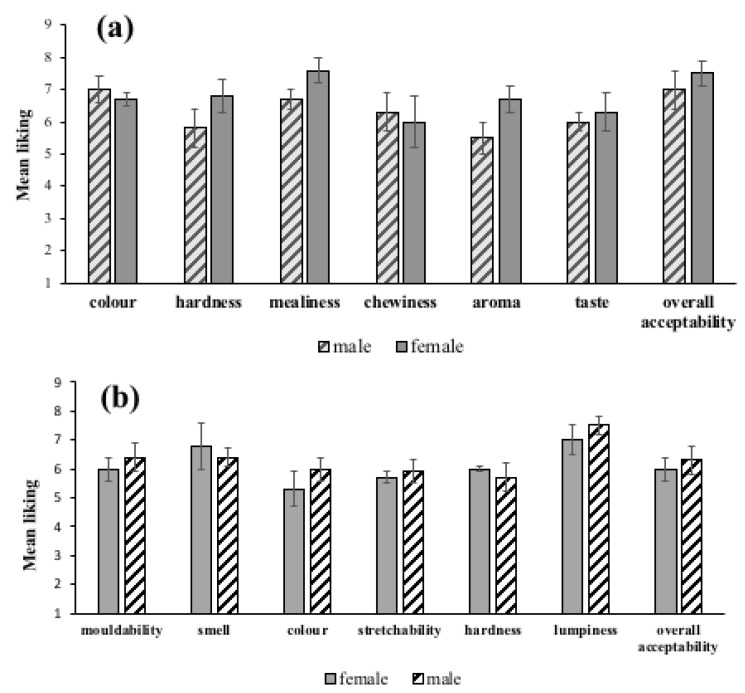
Sex-disaggregated preferences for (**a**) boiled and (**b**) pounded *Dioscorea alata*.

**Figure 4:**
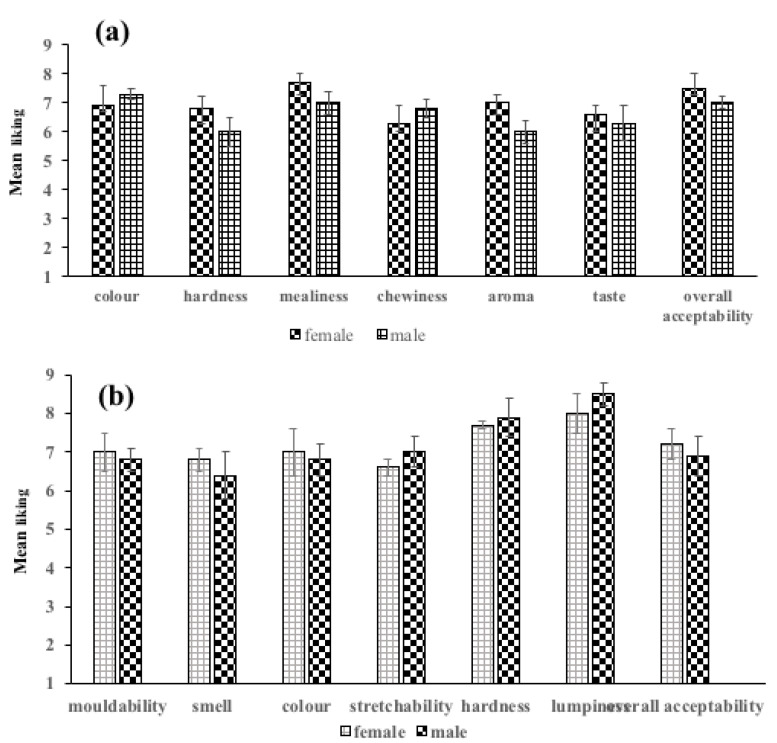
Sex-disaggregated preferences for (**a**) boiled and (**b**) pounded *Dioscorea rotundata*.

The effect of sex of respondents on the preference of descriptors of quality of boiled and pounded yam were determined. As shown in Figure 3a,b and Figure 4a,b, no significant differences were obtained (*p* > 0.05), and in terms of overall liking, no significance was observed (*p* > 0.05). The results point to the fact that males and females like descriptors of quality of boiled and pounded *D. alata* and *D. rotundata* in the same way. Findings from gender-disaggregated interviews conducted in the same communities by Effah-Manu et al. [13] contrast this observation, as preferences showed an association of descriptor preferences with sex when Cramer’s V (Phi) was used. Lombardo et al. [20] observed that sex-specific taste preferences could account for differences in eating behaviour. The difference observed between the consumer test and interview results may be attributed to the number of respondents used (i.e., 120 and 684), respectively, for consumer test and interviews. 

### 3.4. Kruskal–Wallis Test Statistic of the Effects of Age on the Liking of Descriptors of Boiled and Pounded D. alata and D. rotundata Yam Varieties

The Kruskal–Wallis test is a nonparametric test that is useful for more than two independent samples. This test assumes that the observations in each group come from populations with distribution and that the samples are random and independent. A Kruskal–Wallis rank test for differences was employed to determine preferences based on age categories (i.e., <30, 30–65 and >65). The results indicate that sensory perception of boiled and pounded yam varieties do not vary (*p* > 0.05) across the various age groupings (Supplementary Materials Tables S1–S4 V). Similar observations were made in the interviews conducted in the same communities by Effah-Manu et al. [13]. Work performed by Koehler and Leonhaeuser [21] show that age affects consumer preferences.

### 3.5. Multiple Comparison for D. alata Yam Varieties and Descriptors of Boiled and Pounded Yam

**Figure 5:**
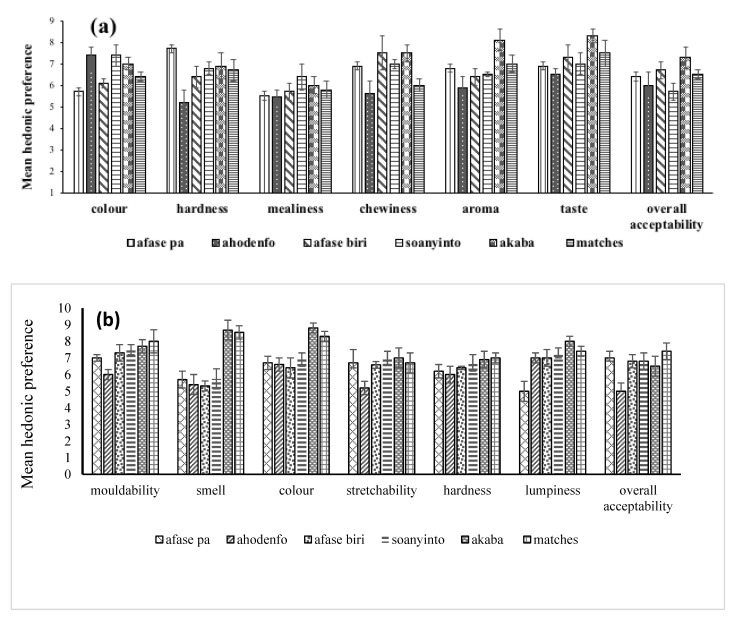
Preference level of quality descriptors of (**a**) boiled and (**b**) pounded *Discorea alata* varieties.

Multiple comparison of boiled yam shows that *akaba* (local *D. alata*) was the variety that significantly differed from the other varieties for most of the descriptors (Figure 5a). CRI *Afase pa* generally varied from the other varieties in colour but had same mealiness with the other varieties. CRI *ahodenfo* varied from the others in mealiness and was different from chewiness, aroma, taste and overall acceptability. CRI *afase biri* varied from *akaba* in all the descriptors and overall acceptability, except hardness and mealiness. The mean taste of *akaba* differed significantly from the varieties. In terms of overall acceptability, *matches* was only significantly different from *ahodenfo*. CRI *afase soanyinto* was different from *akaba* in colour, aroma and taste. *Matches* significantly differed from *afase pa* and *soanyinto* in terms of aroma and colour. The *matches* variety had higher scores for mouldability, aroma and hardness (Figure 5b). *Afase biri* and *soanyinto* are equally good varieties for pounding, as they came second in terms of overall acceptability. The colour and the aroma of these varieties may have accounted for their low ratings. For example, *afase biri*, which has a cocoyam colour, resulted in low preference compared to the other varieties. The consumers commented that “these varieties do not have the usual yam aroma”. 

### 3.6. Multiple Comparison for D. rotundata Yam Varieties and Descriptors of Boiled and Pounded Yam

**Figure 6:**
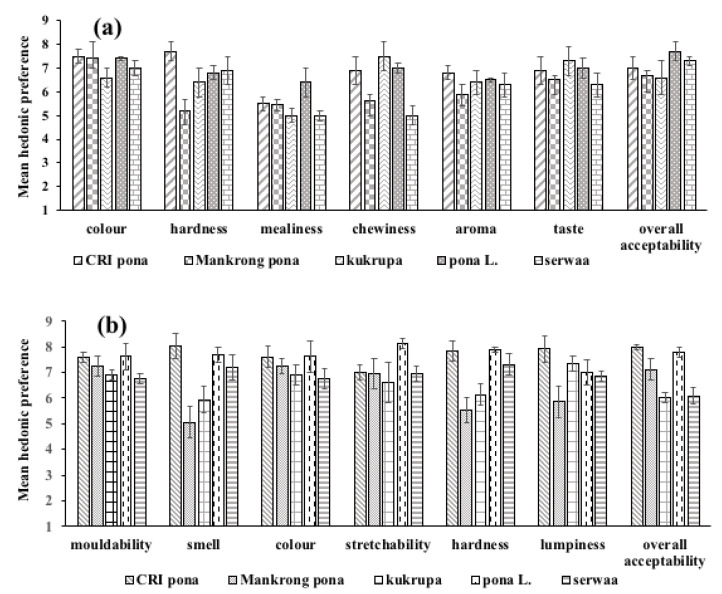
Preference level of quality descriptors of (**a**) boiled and (**b**) pounded *Discorea retundata* varieties.

Multiple comparison for boiled yam from *D. rotundata* varieties varied significantly (*p* < 0.05), as shown in Figure 6a. The most preferred variety was *pona* (local), which varied significantly from all the varieties in all descriptors except the taste of CRI *pona*. CRI *mankrong pona* differed from CRI *pona* in mealiness, hardness, mealiness, chewiness and aroma. *Pona* (local) differed significantly from *mankrong pona* in colour, hardness, mealiness and chewiness and from CRI *pona* in colour, hardness, mealiness, aroma and overall acceptability. CRI *pona* was significantly different in overall acceptability and varied from *mankrong pona* in colour, *pona* (local) in hardness and taste. The aroma and taste of boiled CRI *pona* were liked similarly to the local *pona*. This implies that this variety can compete well on the Ghanaian market.

Generally, some differences existed among the descriptors for the five pounded yam samples (Figure 6b). *Mankrong pona* and *kukrupa* significantly differed in overall acceptability. While *pona* local differed in colour mouldability and lumpiness, *kukrupa* was different from *serwaa* in terms of mouldability and hardness, while *mankrong pona* only varied in springiness from *pona* (local). *Mankrong pona* was similar to CRI *pona* and *pona* (local) in all descriptors and overall acceptability, but varied from *serwaa* and *kukrupa* in acceptability. CRI *kukrupa* was not significantly different from CRI *pona* and *pona* (local) but differed from *serwaa* and *mankrong pona*. CRI *pona* varied from *serwaa* in aroma, colour and lumpiness and from *pona* (local) in all descriptors except aroma. In overall acceptability, *pona* (local) was different from only CRI *pona* but differed from *serwaa* in aroma, colour and lumpiness. Colour and stretchability of CRI *pona* were preferred more than local *pona*.

### 3.7. Descriptive Statistics of Variations in Quality Descriptors of Pounded D. rotundata Varieties

The local *pona* was highly preferred because of its high mealiness compared to the new varieties. Cassava mealiness is associated with high starch content, as determined by Uchendu et al. [22]. Since the CRI *pona* had starch content of 17%, which is close to the local *pona* with starch content of 20%, it could be the reason why the CRI *pona* came second to the local *pona* in terms of mealiness. The low liking of *kukrupa* was due to its hardness. This result correlates with the result of the texture analysis determined by Effah-manu et al. [17], where CRI *kukrupa* variety was the hardest variety, with a value of 4.47 N.

Moreover, the high stretchability of local *pona* made it a preferred variety after CRI *pona*. From the previous work conducted, that these two varieties have approximately the same amylose contents of these varieties could be the reason (CRI *pona*, 16.9%; local *pona*, 17.9%). The amylose content of rice is reported to affect its springiness (stretchability). According to Xu et al. [23], springiness of rice was directly related to amylose content. Heo et al. [24] also worked on noodles and found a significant correlation of springiness to amylose content. 

### 3.8. Relative Importance Index (RII) of D. alata and D. rotundata Yam Varieties

To determine the ranking of the different varieties in terms of overall liking, the relative importance index (RII) was used. Johnson and LeBreton [25] define the RII as the proportionate contribution each predictor makes to R^2^, considering both the unique contribution of each predictor by itself and its incremental contribution when combined with the other predictors. The rankings (Table 3 and Table 4) are therefore directly related to the descriptive statistics.

### 3.9. Sex and Descriptor Preferences for Boiled and Pounded Yam 

To assess for significance in a scale- or ordinal-dependent variable by a single dichotomous independent variable, the Mann–Whitney U test, a nonparametric test, is used, as described by McKnight and Najab [26]. Thus, the effect of sex on descriptor preferences was determined. The results (Supplementary Materials Tables S5 and S6) indicate that no significance exists (*p* < 0.05) between females and males for preferences of all the descriptors. Results from the survey also showed that for boiled yam, there is no significant difference in preference by males and females, but rather, significance was observed for pounded yam descriptors in the work conducted by Effah-Manu et al. [13]. These findings may result from the diversity of respondents involved in both studies. 

### 3.10. Preferences by Urban and Rural Consumers for Quality Descriptors of ampesi and fufu 

Variations in preferences for the various descriptors by urban and rural consumers was analysed (Supplementary Materials Tables S7 and S8). For boiled yam, mean rank for hardness (760.58) differed significantly (*p* < 0.05), with a *p*-value of 0.00 from respondents from the district capitals (680.42). Preference for hardness is lower in the cities than the villages. A *p*-value of 0.004 was also obtained for aroma. The mean rank for aroma is higher in the villages (750.54) than the cities (690.46). This observation could be due to the fact that an accompanying good/tasty sauce for eating *ampesi* is sometimes hard to come by in some rural communities, hence the preference of yams with high aromatic compounds. According to Qin et al. [27] volatile constituents of foods have been used in breeding programs and can be improved to ensure the production of high-aroma yams. 

Preference for descriptors of pounded yam (SD8) aroma, colour and lumpiness differed significantly (*p* < 0.05) between the other communities and the cities. Pounded yam aroma had least mean score of 664.54 compared to 776.46 in the rural communities. A similar observation was made for the descriptor colour, with mean rank values of 757.11 for other communities vs. 683.89 for the district capitals. Both descriptors had a *p*-value of 0.000. Lumpiness was more highly detected by urban consumers than respondents from the rural communities, and was significant, with a *p*-value of 0.001. 

Mean values for boiled *D. rotundata* varieties (Supplementary Marials Table S10) showed significance (*p* < 0.05) for the descriptors of colour and aroma, with mean rank values for the district capitals being higher than the other communities. Differences occurred between the district capitals and the other communities in preferences for colour, mouldability and lumpiness (Supplementary Materials Table S9). The rank values were higher for these descriptors in the rural communities. Thus, these descriptors of the varieties presented were liked/appreciated more in urban communities than rural communities. Generally, rural consumers who mainly grow and consume a lot of *ampesi* and *fufu* may have varying preferences for descriptors as a result of access to different varieties that provide them with a great idea of variations in various descriptors for yam. Conversely, Arsil et al. [28] found that factors that influence consumers’ preferences seemed remarkably homogeneous in terms of food quality, although consumer demographics differed. Generally, the descriptor preferences that varied include aroma, colour, mouldability and lumpiness. These descriptors can, however, be improved in breeding programs for increased acceptability of released varieties.

Overall, rural consumers preferred all the descriptors of boiled *D. rotundata* than urban consumers, whereas urban consumers liked the pounded yam varieties better than the rural consumers.

### 3.11. Limitations of Work

The time frame for consumer tests for the two varieties (*D. alata* and *D. rotundata*) differed due to availability of samples. Because of this limitation, not all the respondents who were involved in the testing of *D. alata* varieties were available for testing *D. rotundata* varieties. Moreover, since the entire testing period lasted for three weeks, the length of storage may have affected some descriptors of quality for communities such as Gulumpe and Kawampe that were last to conduct the testing.

## 4. Conclusions

The findings show that preferences for descriptors of boiled and pounded yam did not vary across different age segmentation. Females and males perceive boiled yam quality descriptors in the same way but varied for pounded yam. The descriptors that influenced the variations in preferences include colour, lumpiness and mouldability. The relative importance index (RII) for the *D. rotundata* varieties showed that they are all good varieties for boiling and pounding. Generally, in terms of urban and rural consumers, new *D. rotundata* yam varieties were highly liked by rural compared to urban consumers. The preference ranks for descriptors of quality of pounded yam from the new varieties by urban consumers show that their availability on the markets will be a good competition for the locally known varieties, which are used for pounding. *D. alata* varieties (local) were still more acceptable than the new CRI varieties due to the aroma. 

## Figures and Tables

**Table 1:** (**a**) Attributes generated by trained sensory panellists for boiled yam; (**b**) attributes generated by trained panellists for pounded yam.

Attribute	Panel Definition	How to Measure	Scale
(**a**)			
Sweet taste	The taste perceived when eating food rich in sugars	Chew and/or swallow to determine the intensity of sweetness	0—bitter 5—bland 10—high sugar intensity like local pona
Smell/aroma	What is perceived in the nose when sample brought close to the nose/after chewing	Bring sample close to the nose/chew and swallow sample for aroma	0—cocoyam/cassava aroma 10—yam aroma
Colour	Degree of whiteness	Visual inspection	0—grey 5—cream 10—white
Hardness	Mechanical textural attribute relating to the force required to achieve a given deformation	Hand: pressing with fingers/taking a morsel Mouth: amount of time used in masticating to softness before swallowing	0—soft like ripe pawpaw 5—slightly ripened avocado pear 10—apple
Mealiness/waxiness	Smoothness or roughness of sample Mealy: ease of disintegration of boiled yam Waxy: extent to which yam remains intact and does not disintegrate easily	Hand feel Mouth feel when masticating	0—not mealy/waxy 10—mealy
Chewiness	A measure of energy required to masticate the food (gumminess × springiness)	Chew sample in the mouth	0—highly chewy 10—less chewy
(**b**)			
Smell	What is perceived in the nose when sample is served/opened	Bring sample close to the nose	0—cocoyam 5—cassava 10—yam aroma
Colour	Degree of whiteness	Visual inspection	0—light pink/grey 5—cream 10—white
Fracturability	Mechanical textural attribute associated with cohesiveness and hardness and with the force necessary to break a product into crumbs or pieces	Feel between the fingers/breaking off/tearing a portion of the sample	0—like cake 10—banku with about 20% cassava dough
Mouldability	Ability to be made into a shape without separating.	By hand: mould sample in the palm and observe if there is disintegration	0—like banku made from only corn dough (etew) 10—cassava fufu
Hardness	Mechanical textural attribute relating to the force required to achieve a given deformation	Hand: pressing with fingers/taking a morsel Throat: swallowing with water	0—very soft TZ 10—cold koose
Lumpiness/consistency	Presence of lumps	Hand feel	0—corn porridge with lumps 10—no lumps
Springiness/ Stretchability	The tendency of sample to return to its original state after it has been stretched/pressed	Stretch/press sample with fingers to determine how fast it will return to original position	0—not springy/stretchy 10—springy/stretchy
Stickiness	Tendency of sample to bind to other surfaces	By mouth: initiate swallowing; sample dipped in water Bowl: visual inspection of sample in bowl/turn slightly to detect stickiness	0—like Ga kenkey 10—cassava fufu

**Table 2:** Average scores for boiled yam from *D. alata* and *D. rotundata* yam varieties.

Yam Variety	Species	Whiteness	Aroma	Sweet Taste	Hardness	Mealiness	Chewiness
CRI *afase ahodenfo*	*D. alata*	3.00	6.08	6.08	4.91	6.92	7.41
CRI *afas biri*	*D. alata*	2.50	4.5	4.375	6.42	6.00	5.25
CRI *afase pa*	*D. alata*	3.5	3.16	4.75	4.5	6.75	5.83
CRI *afase soanyinto*	*D. alata*	2.91	2.91	3.5	5.75	6.5	6.5
*akaba* (local)	*D. alata*	4.41	6.5	5.87	5.08	7.45	6.83
*matches* (local)	*D. alata*	8.00	5.5	5.67	3.08	6.41	6.33
CRI kukrupa	*D. rotundata*	5.67	6.42	5.91	6.58	5.67	5.58
CRI *pona*	*D. rotundata*	5.25	7.08	5.25	6.25	5.75	7.33
CRI *mankrong pona*	*D. rotundata*	4.91	7.5	5.5	5.91	5.58	5.33
*serwaa* (local)	*D. rotundata*	5.66	6.41	5.91	6.58	5.67	5.58
*pona* (local)	*D. rotundata*	6.08	7.00	6.08	4.25	5.91	6.16
**Yam Variety**	**Species**	**Aroma**	**Colour**	**Stretchability**	**Lumpiness**	**Hardness**	**Fracturability**
CRI *afase ahodenfo*	*D. alata*	4.25	2.08	1.41	3.92	3.75	3.25
CRI *afase soanyinto*	*D. alata*	5.00	7.00	1.58	4.58	3.41	4.08
CRI *afase biri*	*D. alata*	5.17	4.16	3.63	4.91	5.66	4.83
CRI *afase pa*	*D. alata*	6.12	3.16	1.17	3.91	3.25	3.83
*akaba* (local)	*D. alata*	6.58	2.41	3.25	4.83	5.5	6.83
*matches* (local)	*D. alata*	7.09	8.09	3.81	5.63	6.36	3.36
CRI *kukrupa*	*D. rotundata*	8.42	7.33	6.00	4.41	7.00	4.42
CRI *pona*	*D. rotundata*	8.91	6.5	4.92	8.5	8.25	6.92
CRI *Mankrong pona*	*D. rotundata*	8.33	5.92	5.41	7.66	7.42	4.75
*serwaa* (local)	*D. rotundata*	8.42	7.33	6.00	4.41	7.00	4.42
*pona* local	*D. rotundata*	8.08	6.25	7.00	8.66	7.66	6.41

Scale 0–10; where 0 is least liked and 10 is highly liked.

**Table 3:** Relative index for choice of yam variety for overall acceptability of boiled and pounded yam (*D. alata*).

Varieties	RII	Ranking	Varieties	RII	Ranking
Boiled				Pounded	
*Akaba*	0.22	1	*Matches*	0.22	1
*Afase biri*	0.25	2	*Soanyinto*	0.24	2
*Matches*	0.28	3	*Akaba*	0.24	2
*Afase pa*	0.28	4	*Afase pa*	0.26	3
*Afase soanyinto*	0.30	5	*Afase Biri*	0.28	4
*Afase ahodenfo*	0.32	6	*Ahodenfo*	0.36	5

**Table 4:** Relative index for choice of yam variety for overall acceptability of boiled and pounded yam (*D. rotundata*).

Varieties	RII	Ranking	Varieties	RII	Ranking
	Boiled			Pounded	
*Local pona*	0.22	1	*CRI pona*	0.22	1
*CRI pona*	0.25	2	*Local pona*	0.24	2
*Mankrong pona*	0.28	3	*Mankrong pona*	0.24	3
*serwaa*	0.28	4	*kukrupa*	0.26	4
*kukrupa*	0.30	5	*serwaa*	0.28	5

## Data Availability

The data supporting the results of this study are all included in the present article.

## Supplementary Materials

The following supporting information can be downloaded at: https://www.mdpi.com/article/10.3390/foods12030537/s1, Table S1. Kruskal Wallis test statistics of the effect of age group liking of D. rotundata boiled yam. Table S2. Kruskal Wallis test statistics of the effect of age group and liking of D. rotundata pound-ed yam. Table S3. Kruskal Wallis test statistics of the effects of age group and liking of D. alata boiled yam. Table S4. Kruskal Wallis test statistics of the effect of age group and liking of D. alata pounded yam. Table S5. Descriptive statistics of sex differences in attribute preference for boiled yam. Table S6. Descriptive statistics of sex differences in attribute preference for boiled yam. Table S7. Mann-Whitney U & Wilcoxon test of communities and their descriptors of boiled D. alata yam varieties. Table S8. Mann-Whitney U & wilcoxon test of communities and their descriptors of pounded D. alata varieties. TableS9. Mann-Whitney test of the effects of district capitals and other communities on the level of liking for pounded D. rotundata varieties. Table S10. Mann-Whitney test of the effects of district capitals and other communities on the level of liking for boiled D. rotundata varieties.
