# Supplementary material for: Gender-Disaggregated Consumer Testing and Descriptive Sensory Analysis of Local and New Yam Varieties
Source: Foods. 2023 Jan 25;12(3):537. doi: 10.3390/foods12030537 (PMC9914015; doi:10.3390/foods12030537)
Supplement: Supplementary file 1 [file foods-12-00537-s001.zip › foods-2113247-supplementary.pdf]

foods

MDPI

Supplementary Materials

Table S1. Kruskal Wallis test statistics of the effect of age group liking of *D. rotundata* boiled yam.

	Ranks			Kruskal Wallis Test Statistics		
	Age Groupings	N	Mean Rank	Chi-Square	Df	*P*-Value
Colour	< 30 years	323	604.43	1.548	2	0.461
	30-65 years	817	595.54			
	> 65 years	60	646.92			
Hardness	< 30 years	323	611.45	0.501	2	0.778
	30-65 years	817	596.31			
	> 65 years	60	598.55			
Mealiness	< 30 years	323	605.08	0.400	2	0.819
	30-65 years	817	597.12			
	> 65 years	60	621.86			
Chewiness	< 30 years	323	614.53	3.608	2	0.165
	30-65 years	817	600.24			
	> 65 years	60	528.44			
Aroma	< 30 years	323	611.88	0.543	2	0.762
	30-65 years	817	596.29			
	> 65 years	60	596.57			
Taste	< 30 years	323	585.83	1.068	2	0.586
	30-65 years	817	607.07			
	> 65 years	60	590.04			
Overall acceptability	< 30 years	323	620.45	2.877	2	0.237
	30-65 years	817	596.54			
	> 65 years	60	547.08			

Table S2. Kruskal Wallis test statistics of the effect of age group and liking of *D. rotundata* pounded yam.

	Ranks			Kruskal Wallis Test Statistics		
	Age Groupings	N	Mean Rank	Chi-Square	Df	*P*-Value
**Aroma**	< 30 years	325	590.73	1.740	2	0.419
	30-65 years	815	600.63			
	> 65 years	60	651.71			
	Total	1200				
**Colour**	< 30 years	325	622.16	2.215	2	0.330
	30-65 years	815	593.84			
	> 65 years	60	573.67			
	Total	1200				
**Mouldability**	< 30 years	325	613.15	1.175	2	0.556
	30-65 years	815	593.71			
	> 65 years	60	624.13			
	Total	1200				
**Stretchability**	< 30 years	325	590.79	0.395	2	0.821
	30-65 years	815	603.77			
	> 65 years	60	608.71			
	Total	1200				
**Hardness**	< 30 years	325	619.59	1.746	2	0.418
	30-65 years	815	591.85			

	> 65 years	60	614.62			
	Total	1200				
**Lumpiness**	< 30 years	325	602.37	1.038	2	0.595
	30-65 years	815	596.75			
	> 65 years	60	641.37			
	Total	1200				
**Overall acceptability**	< 30 years	325	612.22	0.550	2	0.760
	30-65 years	815	596.37			
	> 65 years	60	593.06			
	Total	1200				

Table S3. Kruskal Wallis test statistics of the effects of age group and liking of *D. alata* boiled yam.

Ranks				Test Statistics		
Age category		N	Mean Rank	Chi-Square	Df	*P*-Value
Colour	< 30 years	343	694.97	2.831	2	0.243
	30-65 years	1037	725.82			
	> 65 years	60	774.45			
	Total	1440				
Hardness	< 30 years	343	719.54	0.778	2	0.678
	30-65 years	1037	723.33			
	> 65 years	60	677.07			
	Total	1440				
Mealiness	< 30 years	343	699.00	1.977	2	0.372
	30-65 years	1037	724.93			
	> 65 years	60	766.93			
	Total	1440				
Chewiness	< 30 years	343	690.44	3.700	2	0.157
	30-65 years	1037	727.06			
	> 65 years	60	778.93			
	Total	1440				
Aroma	< 30 years	343	679.37	5.872	2	0.053
	30-65 years	1037	730.69			
	> 65 years	60	779.43			
	Total	1440				
Taste	< 30 years	343	711.97	1.001	2	0.606
	30-65 years	1037	720.66			
	> 65 years	60	766.43			
	Total	1440				
Overall acceptability	< 30 years	343	720.77	0.023	2	0.988
	30-65 years	1037	719.98			
	> 65 years	60	728.00			
	Total	1440				

Table S4. Kruskal Wallis test statistics of the effect of age group and liking of *D. alata* pounded yam.

Descriptors	Age	Ranks		Test Statistics		
		N	Mean Rank	Chi-Square	Df	P-Value
Aroma	< 30 years	310	723.09	0.020	2	0.990
	30-65 years	1010	719.67			
	> 65 years	120	720.78			
	Total	1440				

Colour	< 30 years	310	737.81	1.409	2	0.494
	30-65 years	1010	712.99			
	> 65 years	120	739.01			
	Total	1440				
Stretchability	< 30 years	310	705.51	0.726	2	0.695
	30-65 years	1010	726.21			
	> 65 years	120	711.18			
	Total	1440				
Hardness	< 30 years	310	695.57	1.968	2	0.374
	30-65 years	1010	729.80			
	> 65 years	120	706.64			
	Total	1440				
Lumpy	< 30 years	310	735.94	4.745	2	0.093
	30-65 years	1010	709.85			
	> 65 years	120	770.22			
	Total	1440				
Overall acceptability	< 30 years	310	724.52	0.571	2	0.752
	30-65 years	1010	722.32			
	> 65 years	120	694.77			
	Total	1440				

Table S5. Descriptive statistics of sex differences in attribute preference for boiled yam.

Boiled Yam							
**Descriptors**	**Sex**	**Descriptive**			**F-Statistics**		**Significance**
		**N**	**Mean**	**Std. Dev.**	**F**	**Sig.**	
Colour	Male	704	2.60	2.403	0.141	0.708	No Significance
	Female	736	2.55	2.435			
	Total	1440	2.58	2.419			
Hardness	Male	704	2.53	2.165	0.67	0.413	No Significance
	Female	736	2.63	2.356			
	Total	1440	2.58	2.264			
Mealiness	Male	704	2.31	1.846	0.841	0.359	No Significance
	Female	736	2.4	1.979			
	Total	1440	2.36	1.915			
Chewiness	Male	704	2.24	1.881	0.686	0.408	No Significance
	Female	736	2.32	2.061			
	Total	1440	2.28	1.975			
Aroma	Male	704	2.61	2.398	0.972	0.324	No Significance
	Female	736	2.49	2.336			
	Total	1440	2.55	2.366			
Taste	Male	704	2.29	1.978	0.42	0.517	No Significance
	Female	736	2.36	2.121			
	Total	1440	2.33	2.052			
Overall Acceptability	Male	704	2.48	1.925	0.041	0.839	No Significance
	Female	736	2.46	1.978			
	Total	1440	2.47	1.952			

Table S6. Descriptive statistics of sex differences in attribute preference for boiled yam.

Pounded Yam							
Dependent variables	Gender	Descriptive			F-Statistics		Significance
		N	Mean	Std. Dev.	F	Sig.	
Smell	Male	702	2.21	2.098	0.248	0.618	No Significance
	Female	738	2.16	1.987			
	Total	1440	2.18	2.042			
Colour	Male	702	2.02	1.873	0.71	0.399	No Significance
	Female	738	2.11	2.043			
	Total	1440	2.06	1.962			
String_Elastic	Male	702	2.4	1.918	0.296	0.587	No Significance
	Female	738	2.35	1.816			
	Total	1440	2.38	1.866			
Hard_Soft	Male	702	2.29	1.875	0.194	0.66	No Significance
	Female	738	2.25	1.697			
	Total	1440	2.27	1.786			
Sticky	Male	702	2.04	1.662	1.346	0.246	No Significance
	Female	738	1.94	1.405			
	Total	1440	1.99	1.536			
Lumpy	Male	702	1.65	1.539	2.439	0.119	No Significance
	Female	738	1.54	1.231			
	Total	1440	1.59	1.39			
Overall Ac-ceptability	Male	702	2.37	1.897	0.017	0.897	No Significance
	Female	738	2.39	1.873			
	Total	1440	2.38	1.884			

Table S7. Mann-Whitney U & Wilcoxon test of communities and their descriptors of boiled *D. alata* yam varieties.

Descriptor	Communities	Ranks		Mann-Whitney U & Wilcoxon Test			
		N	Mean Rank	Mann-Whitney U	Wilcoxon W	Z	*P*-Value
Colour	Other communities	720	738.93	245929	505489	-1.800	0.072
	District capital	720	702.07				
	Total	1440					
Hardness	Other communities	720	760.58	230342	489902	-3.844	0.000
	District capital	720	680.42				
	Total	1440					
Mealiness	Other communities	720	717.25	256858	516418	-.313	0.755
	District capital	720	723.75				
	Total	1440					
Chewiness	Other communities	720	724.50	256318	515878	-.391	0.696
	District capital	720	716.50				
	Total	1440					
Aroma	Other communities	720	750.54	237574	497134	-2.917	0.004
	District capital	720	690.46				
	Total	1440					
Taste	Other communities	720	712.60	253509	513069	-.771	0.441
	District capital	720	728.40				
	Total	1440					

Overall Acceptability	Other communities	720	728.44	253481	513041	-.757	0.449
	District capital	720	712.56				
	Total	1440					

Table S8. Mann-Whitney U & wilcoxon test of communities and their descriptors of pounded *D. alata* varieties.

Descriptor	Communities	Ranks		Mann-Whitney U & Wilcoxon Test			
		N	Mean Rank	Mann-Whitney U	Wilcoxon W	Z	*P*-Value
Aroma	Other communities	720	776.46	218910	478470	-5.642	.000
	District capital	720	664.54				
	Total	1440					
Colour	Other communities	720	757.11	232839	492399	-3.773	.000
	District capital	720	683.89				
	Total	1440					
Stretchability	Other communities	720	748.70	238898	498458	-2.714	.007
	District capital	720	692.30				
	Total	1440					
Hard/Soft	Other communities	720	724.92	256017	515577	-.427	.669
	District capital	720	716.08				
	Total	1440					
Lumpy	Other communities	720	747.84	239519	499079	-3.244	.001
	District capital	720	693.16				
	Total	1440					
Overall Acceptability	Other communities	720	739.81	245295	504855	-1.868	.062
	District capital	720	701.19				
	Total	1440					

TableS9. Mann-Whitney test of the effects of district capitals and other communities on the level of liking for pounded *D. rotundata* varieties.

		N	Mean Rank	Mann-Whitney U	Wilcoxon W	Z	*P*-Value
Aroma	District capital	600	596.70	177717.50	358017.50	-0.401	0.689
	Other communities	600	604.30				
	Total	1200					
Colour	District capital	600	593.93	176058.50	356358.50	-0.704	0.482
	Other communities	600	607.07				
	Total	1200					
Mouldability	District capital	600	622.75	166649.00	346949.00	-2.382	0.017
	Other communities	600	578.25				
	Total	1200					
Strechability	District capital	600	619.30	168720.00	349020.00	-1.966	0.049
	Other communities	600	581.70				
	Total	1200					
Hardness	District capital	600	604.02	177888.50	358188.50	-0.368	0.713

	Other communities	600	596.98				
	Total	1200					
Lumpiness	District capital	600	619.70	168479.00	348779.00	-2.017	0.044
	Other communities	600	581.30				
	Total	1200					
Overall Acceptability	District capital	600	611.25	173552.50	353852.50	-1.110	0.267
	Other communities	600	589.75				
	Total	1200					

Table S10. Mann-Whitney test of the effects of district capitals and other communities on the level of liking for boiled *D. rotundata* varieties.

	**Communities**	**N**	**Mean Rank**	**Mann-Whitney U**	**Wilcoxon W**	**Z**	***P*-value**
Colour	District capital	600	568.37	139280.5	264530.5	-2.239	0.025
	Other communities	600	529.06				
	Total	1200					
Hardness	District capital	600	559.35	144691.5	269941.5	-1.076	0.282
	Other communities	600	539.88				
	Total	1200					
Mealiness	District capital	600	538.98	143085.5	323385.5	-1.386	0.166
	Other communities	600	564.33				
	Total	1200					
Chewiness	District capital	600	563.44	142236.5	267486.5	-1.59	0.112
	Other communities	600	534.97				
	Total	1200					
Aroma	District capital	600	563.23	142363	267613	-1.554	0.12
	Other communities	600	535.23				
	Total	1200					
Taste	District capital	600	567.14	140013.5	265263.5	-2.038	0.042
	Other communities	600	530.53				
	Total	1100					
Overall Acceptability	District capital	600	556.84	146196.5	271446.5	-0.762	0.446
	Other communities	600	542.89				
	Total	1100					
